# The Use of MALDI-TOF Mass Spectrometry to Analyze Commensal Oral Yeasts in Nursing Home Residents

**DOI:** 10.3390/microorganisms9010142

**Published:** 2021-01-09

**Authors:** Jang-Jih Lu, Hsiu-Jung Lo, Chih-Hua Lee, Mei-Jun Chen, Chih-Chao Lin, Yin-Zhi Chen, Ming-Horng Tsai, Shao-Hung Wang

**Affiliations:** 1 Department of Laboratory Medicine, Chang-Gung Memorial Hospital Linkou, Taoyuan City 333, Taiwan; jjlpcp@adm.cgmh.org.tw (J.-J.L.); josh60708@cgmh.org.tw (C.-H.L.); may95173@cgmh.org.tw (M.-J.C.); 2 Department of Medical Biotechnology and Laboratory Science, Chang Gung University, Taoyuan City 333, Taiwan; 3 Department of Medicine, College of Medicine, Chang Gung University, Taoyuan City 333, Taiwan; 4 National Institute of Infectious Diseases and Vaccinology, National Health Research Institutes, Miaoli County 350, Taiwan; hjlo@nhri.edu.tw (H.-J.L.); chihchao.lin@opnano.com (C.-C.L.); injue0705@nhri.edu.tw (Y.-Z.C.); 5 School of Dentistry, China Medical University, Taichung City 404, Taiwan; 6 Division of Neonatology and Pediatric Hematology/Oncology, Department of Pediatrics, Chang Gung Memorial Hospital, Yunlin County 638, Taiwan; mingmin.tw@yahoo.com.tw; 7 Department of Microbiology, Immunology and Biopharmaceuticals, National Chiayi University, Chiayi City 600, Taiwan

**Keywords:** MALDI-TOF MS, commensal yeast, oral cavity microorganisms, CHROMagar *Candida*, geriatric medicine

## Abstract

Matrix-assisted laser desorption ionization time-of-flight mass spectrometry (MALDI-TOF MS) is a rapid and accurate method to identify microorganisms in clinical laboratories. This study isolates yeast-like microorganisms in the oral washes that are collected from non-bedridden nursing home residents, using CHROMagar *Candida* plates, and identifies them using Bruker MALDI-TOF MS. The ribosomal DNA sequences of the isolates are then examined. Three hundred and twenty yeast isolates are isolated from the oral washes. *Candida* species form the majority (78.1%), followed by *Trichosporon/Cutaneotrichosporon* species (8.8%). Bruker MALDI-TOF MS gives a high-level confidence, with a log(score) value of ≥1.8, and identifies 96.9% of the isolates. There are six inconclusive results (1.9%), and those sequences are verified as rare clinical species, including *Candida ethanolica*, *Cutaneotrichosporon jirovecii*, *Exophiala dermatitidis*, and *Fereydounia khargensis*. Almost all of the isolates have a regular color on the CHROMagar *Candida* plates. If the colonies are grouped by color on the plates, a specific dominant yeast species is present in each color group, except for purple or orange isolates. In conclusion, MALDI-TOF MS is verified as a fast, accurate and practical method to analyze oral yeasts in elderly subjects.

## 1. Introduction

Matrix-assisted laser desorption ionization time-of-flight mass spectrometry (MALDI-TOF MS) is a rapid and accurate method to identify microorganisms in a clinical microbiology laboratory [1,2,3]. The process compares the proteomic fingerprint of an unknown microorganism, which mainly consists of ribosome proteins, to those in a reference spectral database. The peak profiles that are generated by Bruker MALDI-TOF MS are matched to reference libraries using the integrated patterns matching algorithm, BioTyper software (Bruker Daltonics, Bremen, Germany). This gives an arbitrary score value of 0 to 3.0 to represent the similarity between the sample and the reference spectrum. A log(score) of ≥2.0 represents successful identification of a species, and a score of 1.7 to 2.0 is acceptable for the genus level. Scores higher than 1.8 represent highly-accurate yeast identification [4,5]. Cultivation is inevitable if microorganisms are identified by MALDI-TOF MS, but the process only requires a minute number of cells, and much less culture time. Along with the development of microbial culturomics, MALDI-TOF MS plays an effective complementary role in microbiome analysis using metagenomics [6,7].

The elderly population (60 years or over) is estimated by the WHO to grow from 12.7% in 2017 to 21.3% in 2050 globally, and from 12.2% to 24.2% in Asia [8]. Therefore, the healthcare needs of elderly people are increasingly significant. A recent study on salivary microbiota in the elderly shows that general frailty is associated with composition and formulation of oral microbiota [9]. More than 100 trillion microbial cells are estimated to inhabit a healthy human body [10]. In the oral cavity, microbes live in the biofilm on oral tissues to protect against environmental stress and host immunity [11]. A dysbiotic imbalance of the indigenous oral microbial community is a cause of oral diseases, including early childhood caries, HIV-associated periodontitis, and rheumatoid arthritis [12,13,14]. A connection between distinct oral microbiota has also been observed in patients with oropharyngeal squamous cell carcinoma [15].

The oral microbiota is a complex community that is primarily composed of bacteria and fungi. Oral mycobiota are more diverse and complex than any other body site in healthy humans [16]. The intake of environmental fungi through food and breathing undoubtedly contributes to the greater fungal diversity in the oral cavity [17]. Thirteen taxa, including the prevalent *Candida* species, *Cladosporium*, *Aureobasidium*, *Saccharomycetales*, *Aspergillus*, *Fusarium*, and *Cryptococcus*, are the core mycobiota in healthy individuals [18]. However, oral mycobiota was observed to change if the host immunity changes: *Candida* species are particularly dominant in immunosuppressed solid organ transplant recipients [16]. In terms of the interaction dynamic between resident fungi and the innate immunity, it is worth examining the salivary mycobiota, especially the yeasts, of non-bedridden elderly subjects.

Mycobiota dysbiosis is an important factor in oral diseases, so a practical method to analyze the changes in yeast populations that are collected from saliva or oral washes is necessary. Chromogenic agar is a practical and effective tool to isolate common yeast in clinical specimens [19,20], but the reported specificity is 62% for bronchial secretion samples [21]. The color and morphology of colonies that are isolated on CHROMagar *Candida* is critical to the presumptive identification of clinically-important *Candida* species [12,21,22]. It is difficult to differentiate non-*Candida* yeasts using CHROMagar *Candida* plates, but the process allows the rapid detection of common *Candida* species in mycobiota [2,5].

CHROMagar *Candida* allows a presumptive identification of common *Candida* species, but the diversity of yeast in oral washes extends far beyond the genus *Candida*. A recent study by the authors demonstrated that *Candida albicans* is majorly cultured from oral washes in a high percentage (52 of 204, 25.5%) of nursing residents, but half of the residents (106/204, 52%) have at least one non-*C. albicans* yeast species [22]. Metagenomics with ribosomal DNA amplification is widely used to characterize of microbiota or mycobiota. MALDI-TOF MS is a rapid and accurate method to identify microorganisms in clinical laboratories [1,2,3]. This study compared different approaches to characterize all the oral yeast isolates, which are single-colony purified by CHROMagar *Candida* from oral washes that are collected from non-bedridden elderly subjects in nursing care institutions [22]. The results demonstrate that the Bruker MALDI-TOF MS is an effective and highly specific method for the identification of oral yeast.

## 2. Materials and Methods

### 2.1. Oral Yeast Isolates

**Figure 1:**
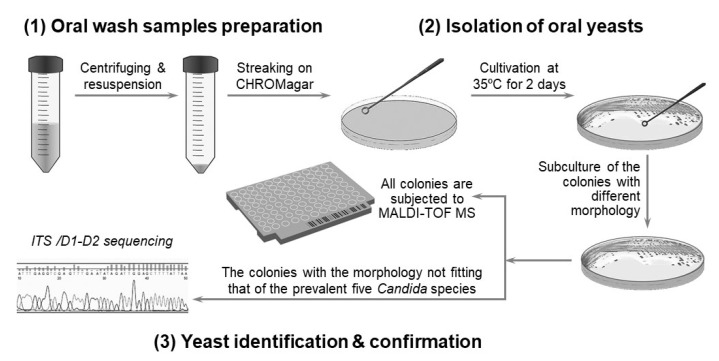
Scheme for the identification of oral yeast for the study. The strategy to isolate and identify yeast involves: (1) preparation of oral wash samples, (2) yeast isolation, and (3) identification and confirmation of the yeast.

This study was approved by the Human Experiment and Ethics Committee of the National Health Research Institute (EC1040411-E). A total of 204 residents of ≥50 years (average age: 77.1; 153 individuals >70 years old) in 10 nursing homes in central Taiwan were enrolled, after giving informed consent. The sampling method has been reported previously [22]. Figure 1 shows the 20 mL oral rinses containing saline that were collected from each person. These rinses were then centrifuged and resuspended in 1 mL of sterile saline. A total of 50 μL of suspension of each sample was streaked onto a CHROMagar *Candida* plate (CHROMagar Company, Paris, France) and incubated at 35 °C for 2 days. If present, colonies from each plate were selected for further analysis. Yeast colonies were identified according to the manufacturer’s instructions or the description of the morphology by Odds and Bernaerts [23].

### 2.2. Bruker MALDI-TOF MS

A full loop of colonies was selected and suspended in 300 μL of deionized water containing 900 μL of absolute ethanol. Following centrifugation, the pellet was then dissolved in 50 μL of 70% formic acid and mixed in a vortex with 50 μL of acetonitrile. The samples were centrifuged, and 1 μL of the clear supernatant was spotted onto the MALDI target plate and air-dried at room temperature. Each spot was then overlaid with 1 μL of CHCA and completely air-dried before the Bruker MALDI-TOF MS measurement. The microorganism was identified, and data analyses were performed using the Bruker LT microflex MALDI-TOF MS (Bruker Daltonics, Bremen, Germany) with Bruker BioTyper 3.0 system software (Bruker Daltonics) [24,25]. A MALDI Biotyper log(score) value >2.0 represents reliable identification at the genus level and a score <1.7 represents ambiguous identification [4,5]. 

### 2.3. Sequence Analysis of Ribosomal DNA

The genomic DNA of yeast was extracted and internal transcribed spacers (ITS) or D1/D2 amplification for identifications was used, as previously described [22,26]. The thermal cycling parameters were an initial denaturation for 5 min at 95 °C and 30 cycles of 1 min at 95 °C, 1 min at 55 °C, and 1 min at 72 °C. A final extension at 72 °C for 10 min was performed at the end of the amplification. The amplicons were purified and then sequenced by the Genomic Medicine Research Core Laboratory of Chang Gung Memorial Hospital Genomic Medicine Core Laboratory, Chang Gung Memorial Hospital. The nucleotide sequence results were analyzed using BLAST searches with default settings against the nonredundant (NR) database at NCBI, the ISHAM-ITS database, and the Candida Genome Database (CGD). The best BLAST hit for the query sequence of each amplicon was used to identify the isolates.

### 2.4. Statistic Analysis

An unpaired Student’s *t*-test was used to compare the value of the Biotyper score: a *p* value < 0.05 was considered to be significant.

## 3. Results

### 3.1. Isolation and Identification of Oral Yeast

**Figure 2:**
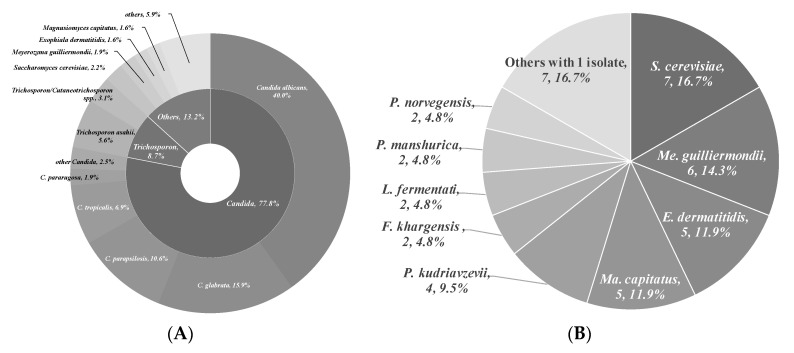
Distribution of commensal oral yeasts in the residents of nursing homes. All identified yeasts were grouped together into taxa and sorted according to population size (**A**). The yeast isolates in the group “Others” do not belong to the genus *Candida* or *Trichosporon/Cutaneotrichosporon*. Details are shown in (**B**).

To isolate and identify the oral yeast isolates of non-bedridden elderly residents in nursing homes, the oral wash samples were collected and cultivated on CHROMagar *Candida* plates. Isolates with different morphologies on each plate were collected, and 320 yeast-like isolates were collected in total. All the fresh colonies of those isolates were subjected to Bruker MALDI-TOF MS (Figure 1). The genus, *Candida* (250 isolates, 78.1%), comprised the majority, followed by *Trichosporon/Cutaneotrichosporon* (28 isolates, 8.8%) (Figure 2 and Table 1). *C. albicans* was the most abundant (129 isolates, 40.3%) and *Trichosporon asahii* (18 isolates, 5.6%) was the largest species of the non-*Candida* isolates. In the non-*Candida*/non-*Trichosporon* group, the probiotic yeast *Saccharomyces cerevisiae* (7 isolates), accounted for 19.4% of the isolates in the rare species of yeasts, followed by *Meyerozyma guilliermondii* (6 isolates; anamorph, *C. guilliermondii*), and others (Figure 2 and Table 1).

### 3.2. Accuracy of Bruker MALDI-TOF MS Identification

Of the 320 yeast isolates, only six isolates could not be positively identified by Bruker MALDI-TOF MS with the BioTyper system. All yeast isolates were further ITS- or D1/D2-sequence verified, unless the colony color and morphology fully matched the patterns of the five major *Candida* species on CHROMagar *Candida*. A total of 302 isolates had log(score) values of more than 2.0, which represents accurate identification. Of the remaining isolates with a log(score) value of more than 1.8, six isolates showed the same results for Bruker MALDI-TOF MS and ITS-sequencing, and two were identified using Biotyper as *Candida guilliermondii* var. *membranifaciens*, which matches the corresponding anamorph *Kodamaea ohmeri* that was identified by ITS sequencing [27]. One isolate was identified using Biotyper as *Candida fermentati*, with a low log(score) value of 1.707, which is the anamorph of the ITS-sequencing result *Meyerozyma caribbica*. In summary, 314 of 320 isolates were accurately identified by the Bruker MALDI-TOF MS Biotyper system. The main spectrum patterns of common *Candida* species (*C. albicans*, *C. glabrata*, *C. parapsilosis*, and *C. tropicalis*) and *Trichosporon asahii* were analyzed individually (Figures S1–S5), and the results show some highly conservative peaks that could be used as biomarker patterns for dominant yeasts in oral cavity. Only six oral wash isolates were untypable, including two each of *Exophiala dermatitidis* and *Fereydounia khargensis*, and one each of *Candida ethanolica* and *Cutaneotrichosporon jiroveci* (formerly *Trichosporon jiroveci*) (Table 2), which were identified by ITS or D1/D2 sequencing. *Candida ethanolica* and *Fereydounia khargensis* are not yet included in the MALDI Biotyper database version 3, which was used for this study. The results convincingly show that Bruker MALDI-TOF MS allows the effective identification of common or rare commensal, environmental yeast species of oral yeast.

### 3.3. Rapid Differentiation of Yeasts by CHROMagar Candida

CHROMagar *Candida* plates were used to screen yeast-like colonies for this study. The color and morphology of the colonies that were isolated on CHROMagar *Candida* are critical for the presumptive identification of clinically-important *Candida* species [20,23,28]. According to the results using Bruker MALDI-TOF MS, 311 of all 320 isolates (97.5%) on CHROMagar *Candida* had a color that matched the reported color [20,29,30,31,32,33]. Most *Candida* species, especially the prevalent *C. albicans* (100%), *C. glabrata* (100%), *C. parapsilosis* (94.1%), and *C. tropicalis* (90.9%), very closely matched the color corresponding to the species on CHROMagar (Table 3), as did non-*Candida* yeasts, such as *T. asahii* (100%) (Table 4).

For all color-matched isolates, six color groups were identified: green, purple/pink, blue, white, gray, and orange. Almost all green colonies were *C. albicans* (129/130 isolates, 99.2%). *C. parapsilosis* accounted for more than 80% of the white group, and *E. dermatitidis* accounted for more than 70% of the gray colonies. In the blue color group, *C. tropicalis* (20/42 isolates, 47.6%) and *T. asahii* (18/42, 42.9%) were the main two species. In the purple/pink color group, there were 15 species, including fifty-one of *C. glabrata*, seven of *S. cerevisiae*, six of *Cutaneotrichosporon mucoides* (formerly *Trichosporon mucoides*), five of *M. capitatus*, four each of *C. krusei* (teleomorph *Pichia kudriavzevii*), *C. pararugosa*, and *Me. guilliermondii* (anamorph *C. guilliermondii*), two of *P. manshurica*, and one each of *C. fermentati*, *C. intermedia*, *C. ethanolica*, *C. metapsilosis*, *C. utilis* (teleomorph, *Cyberlindnera jadinii*), *Clavispora lusitaniae* (anamorph *C. lusitaniae*)**, and *Kluyveromyces marxianus* (anamorph, *C. kefyr*) (Table 5). A wide range of species had a purple/pink color on CHROMagar *Candida*, but the most abundant species was *C. glabrata* (51/90, 56.7%) (Table 5).

## 4. Discussion

### 4.1. Commensal Oral Yeasts in the Elderly

A culture-based mycobiome analysis of oral samples from elderly subjects (age above 65) in New Zealand showed that frequently isolated species included *C. albicans*, *C. glabrata*, *C. parapsilosis*, *Clavispora lusitaniae* (anamorph *C. lusitaniae*), *Meyerozyma guilliermondii* (anamorph, *C. guilliermondii*), *Pichia fermentans*, and *Yarrowia lipolytica* [34]. *P. fermentans* and *Y. lipolytica* were not identified by this study, but a significant population (28/55, 50.9% in non-*Candida* group) was of the group *Trichosporon*, which belongs to human skin commensals and is also frequently found in the mouths of healthy individuals [35]. The very large population of the *Trichosporonaceae* family (including *Trichosporon* and *Cutaneotrichosporon* species) in oral microbiota that were collected from elderly subjects in nursing homes in central Taiwan shows that the *Trichosporon* group is common in the environment. In a study that used samples from one-month old infants, oral mycobiome has a significantly lower alpha diversity than that of skin and anal mycobiomes, which shows that the environment of the oral cavity selects commensal fungi, including *Candida*, *Saccharomyces*, and *Cladosporium*, and that the mycobiome depends more on the environment than the mother’s vaginal mycobiome [36]. *Candida* yeasts are a type of human oral commensal that are mostly located on the tongue [37]. The carriage rate for *C. albicans* in the mouths of healthy individuals is estimated to be 17.7%, but this figure increases to 40.6% in hospitalized individuals [38], so health is a predisposing factor for *C. albicans* colonization in the oral cavity [39]. In addition to the prevalent *C. albicans*, *C. glabrata*, *C. krusei* (teleomorph *P. kudriavzevii*), *C. tropicalis*, and *C. parapsilosis* have been isolated from the oral cavity of healthy individuals [37,39,40]. 

Health professionals should be alerted to the presence of a biofilm that is associated with the *Candida* species, especially in those with reduced antifungal susceptibility. Disorders of the oral cavity are common in senior citizens who have no underlying health conditions, including dental caries, gingivitis, periodontitis, xerostomia, denture stomatitis, and candidiasis [41]. Oral disorders can contribute to poor nutritional status and health and have a negative effect on immunity in the elderly. Dental prostheses also reduce salivary flow and predispose elderly individuals to microbial colonization and biofilm formation, which results in candidiasis.

### 4.2. Limitation of MALDI-TOF MS, Chromogenic Agar, and Ribosomal DNA Sequencing in Microbial Pathogenesis

The results of high-throughput amplicon sequencing and shotgun metagenomics show that there are 12 million species of fungi, of which only 2.2–3.8 million species are reported to be cultivable [42]. There has been a rapid growth in sequence data, and mycobiota/microbiota dysbiosis has been identified as a critical factor in some human diseases [43,44,45]. Some bioinformatics analyses using metagenomics show the importance of some alteration of mycobiota, but pure isolates are required to determine the pathogenesis of candidate pathogens. Ribosomal DNA sequencing and MALDI-TOF MS identification all require pure cultures. Chromogenic agar plates allow better identification in a clinical laboratory because they are selective and differential. CHROMagar *Candida*, which is a presumptive identification method for *C. albicans*, *C. tropicalis*, *C. glabrata*, *C. krusei* (teleomorph *P. kudriavzevii*), and *Trichosporon* spp. [32], does not distinguish many environmental yeasts or some clinically-relevant yeasts. The accumulation of MS profiles for reference fungal isolates means that MALDI-TOF MS is an effective identification method for clinical examination. The Bruker database comprises 604 references for yeast and fungus and includes almost all known pathogenic fungi [46], but mix-cultures are usually misidentified in term of the microorganisms, and only monomicrobial cultures can be specifically identified [47]. In clinical, the limitation of MALDI-TOF MS is to identify different species in a sample with mix species. Recently, mix-microbial identification by MALDI-TOF MS has used specific algorithms to determine the mass spectra biomarkers for microorganisms [47]. Meanwhile, liquid chromatography–tandem MS is also used to identify mix-microbial samples [48,49]. As the mass spectral database grows significantly, yeast identification using MS, including identification of mix-culture, would be expected in the near future.

The limitation of both rDNA sequencing and MALDI-TOF MS is that they identify known species. Even though rDNA sequence is simple, it takes longer preparation time than MALDI-TOF MS. Nevertheless, the MALDI-TOF MS equipment and database are not available to all laboratories. For this study, six isolates cannot be typed using Bruker MALDI-TOF MS, including two yeast species that are not included in the MALDI Biotyper database—one *C. ethanolica* isolate and two *F. khargensis* isolates—and two yeast species that are shown in the database—one *Cu. jiroveci* isolate and two *E. dermatitidis* isolates. That *Cu. jiroveci* isolate was MALDI-TOF MS identified, but its log(score) value was 1.703, which is less than the identification threshold (1.8), is probably due to the limited number of isolates in the database. Five of seven *E. dermatitidis* isolates were identified by MALDI-TOF MS, with a wide range of scores: 2.072, 1.967, 1.951, 1.861, and 1.792, but two were MS untyped. The diversity and polymorphism of *E. dermatitidis* [50] might contribute to the variation in MS patterns.

### 4.3. Mycobiome Analysis Using Culture- and Non-Culture Methods

Advances in DNA sequencing technology mean that many microbiota and mycobiota analyses can be conducted without the need for time-consuming cultivation. However, the oral cavity is the entrance for food, drink, and air, so many environmental microbes or debris can be detected using a non-culture method. This study shows that *Trichosporon* is the second largest genus in the oral cavity of elderly subjects, but it is not widely distributed in samples from a previous ITS-sequencing mycobiome study that was conducted in the Cleveland area on standard Western diets [18]. Oral mycobiota is affected by environment, drink, and food styles, but the difference in yeast taxa for this study and others that use ITS-sequencing is probably due to cultivation. *Malassezia* yeast is a skin commensal yeast that has been reported in oral mycobiota using sequencing methods, but is not detected in oral microorganisms using culture methods, because the fatty acid synthesis pathway is incomplete [51]. Therefore, a cohort study to determine whether the culture-based MALDI-TOF MS method is compatible with analysis using DNA extraction-based sequencing analysis would be of use.

### 4.4. Using of CHROMagar Candida to Differentiate Yeast Species in the Oral Cavity

This study uses chromogenic agar plates to screen yeast-like microorganisms. CHROMagar *Candida* in isolation allows the presumptive diagnosis of clinically-important *Candida* species [20,28,34,37,52]. The colony color and the morphology of the isolates are categorized in Table 5. Of all colony color groups in the oral samples of elderly subjects, green precisely differentiates *C. albicans* from others (129/130 isolates, 99.2%) and white color represents most of the *C. parapsilosis* (32/38 isolates, 84.2%). *C. tropicalis* and *T. asahii* contribute to nearly half of the blue colonies, but the purple or pink colonies show no majority. The color category for this study indeed allows reliable analysis of oral mycobiome.

### 4.5. Use of MALDI-TOF MS to Analyze Oral Mycobiome

**Figure 3:**
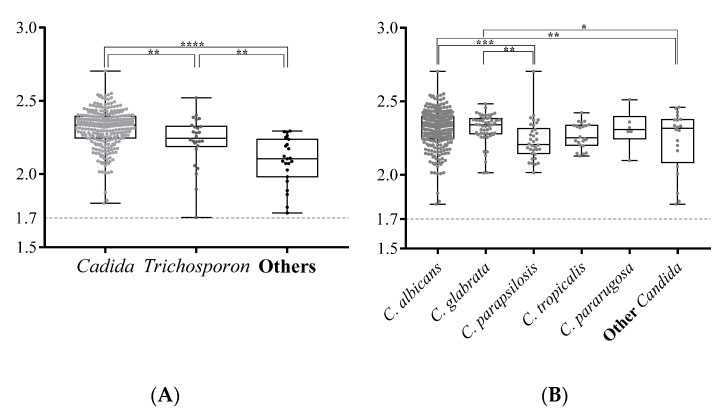
The log(score) values for yeast isolates that are identified using the MALDI-TOF Bruker Biotype. The Biotyper log(score) values for *Candida*, *Trichosporon*, and other genuses are compared in (**A**). All scores for the isolates of the *Candida* species that are identified by this study are compared (**B**). The box plots show the diversity of the log(score) values for the yeast isolates. The upper and lower edges of the boxes correspond to the first and third quartiles. The median is represented by a horizontal line within the box. The whiskers extend from the box in order from the highest to lowest. Each score is marked by a dot. The log(score) values are compared using an unpaired Student’s *t*-test; a *p*-value <0.05 is represented as *, <0.01 as **, <0.001 as ***, and <0.0001 as ****.

The presence of *Candida* strains with reduced susceptibility to antifungal agents, such as reduced susceptibility to azoles in *C. glabrata* and *C. krusei* (teleomorph *P. kudriavzevii*) and reduced susceptibility to flucytosine in *C. krusei* and *C. rugosa*, could pose a problem for immune-compromised elderly subjects who require drug treatment [34]. Therefore, fast and accurate diagnosis of yeast species is important for geriatric medicine. MALDI-TOF MS is a rapid, reliable, economical, and environmentally-friendly method for routine microbial identification. It requires a shorter culture time, because a tiny sample is sufficient, so it can be used to instigate timely and appropriate antibiotic treatment in a clinical setting [3,5]. However, oral mycobiota are highly diverse and contain some rare yeast species, such as *Trichosporon jirovecii* (current name: *Cutaneotrichosporon jirovecii*) [53] and *Lachancea fermentati* [54] (Table 2), which is in contrast with clinical samples. Only species with the morphology of frequently encountered, disease-associated fungi are familiar to physicians or medical technicians, so accurate diagnosis of yeast or molds is difficult. MALDI-TOF MS with a Bruker Biotyper system was used to analyze yeast taxa in the elderly subjects for this study, and a very high identification rate was achieved for oral yeasts (314/320 = 98.1%), with only six yeasts note being untyped. *C. albicans* and *C. glabrata* have a significantly higher Biotyper score (mean score = 2.34 and 2.35 respectively, Figure 3), as well as *Trichosporon* species (mean score = 2.23. This was probably because the Biotyper database contains a large number of MS profiles for *Candida* and *Trichosporon* species. As the number of MS profiles of fungi that can be cultured increases, MALDI-TOF MS will become an increasingly effective means of microbial identification.

## 5. Conclusions

This study used CHROMagar *Candida* to grow oral yeast colonies, which were then identified using Bruker MALDI-TOF MS. This combination better discriminates yeasts that can be cultured, and allows the study of yeasts that are associated with disease. Bruker MALDI-TOF-MS is shown to be a reliable and rapid method for the identification of yeast isolates in the oral washes of elderly residents in nursing homes, especially those of the *Candida* and *Trichosporon* species.

## Figures and Tables

**Table 1:** List of isolated yeasts.

Yeast Isolates	Numbers	Percent
***Candida***	**250**	**78.1%**
*Candida albicans*	129	40.3%
*C. glabrata*	51	15.9%
*C. parapsilosis*	34	10.6%
*C. tropicalis*	22	6.9%
*C. pararugosa*	6	1.9%
other *Candida*	8	2.5%
**Non-*Candida***	**70**	**21.9%**
*Trichosporon asahii*	18	5.6%
*Trichosporon/Cutaneotrichosporon*	10	3.1%
*Saccharomyces cerevisiae*	7	2.2%
*Meyerozyma guilliermondii*	6	1.9%
*Exophiala dermatitidis*	5	1.6%
*Magnusiomyces capitatus*	5	1.6%
*others*	19	5.9%

**Table 2:** Oral yeasts in the residents of nursing homes.

MALDI-TOF MS	Isolate Number	Species
**BioTyper Score ≥ 2.0**	**302**	*C. albicans*, *C. dubliniensis*, *C. glabrata*, *C. intermedia*, *C. krusei*, *C. metapsilosis*, *C. orthopsilosis*, *C. parapsilosis*, *C. pararugosa*, *C. tropicalis*, *Cl. lusitaniae*, *Cr. neoformans*, *Cu. mucoides*, *Cy. jadinii*, *E. dermatitidis*, *K. marxianus*, *Lo. elongisporus, Ma. capitatus*, *Me. guilliermondii*, *P. manshurica*, *P. norvegensis*, *R. mucilaginosa*, *R. toruloides*, *S. cerevisiae*, *T. asahii*, *T. faecale*
**2.0 > BioTyper Score between ≥ 1.8**	**8**	
*Agree with ITS sequencing results*		
Agree with morphology on CHROMagar	5	*C. orthopsilosis*, *E. dermatitidis*, *S. cerevisiae*, *T. faecale*
Disagree with morphology on CHROMagar	1	*Pichia manshurica*
*Disagree with ITS sequencing results*		
Agree with morphology on CHROMagar	2	*Me. guilliermondii* var. *membranifaciens* (ITS: *Kodamaea ohmeri*)
Disagree with morphology on CHROMagar	0	
**BioTyper Score < 1.8**	**4**	
*Agree with ITS sequencing results*		
Agree with morphology on CHROMagar	1	*Cu. jirovecii*
No CHROMagar morphology information available	2	*La. fermentati*
*Disagree with ITS sequencing results*		
No CHROMagar morphology information available	1	*C. fermentati* (teleomorph *Meyerozyma caribbica*)

**Table 3:** Appearance of *Candida* species ^1^ in the oral samples on CHROMagar *Candida* plates.

*Candida* Species	Isolates (Total 320)	Morphology	Colony Color	Frequency of Regular Type
*Candida albicans*	129 (40.3%)	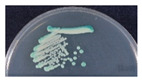	Light green	100% (129/129)
*Candida glabrata*	51 (15.9%)	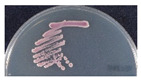	Purple	100.0% (51/51)
*Candida parapsilosis*	34 (10.6%)	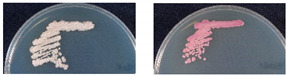	White (regular) Purple (few)	94.1% (32/34)
*Candida tropicalis*	22 (6.9%)	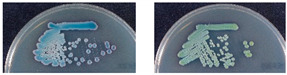	Metallic blue (regular) Dark green (few)	90.9% (20/22)
*Candida guilliermondii*	6 (1.9%)	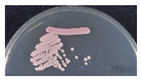	Light purple	100% (6/6)
*Candida pararugosa*	6 (1.9%)	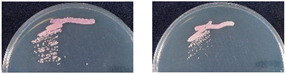	Purple (regular) Pale pink (few)	66.7% (4/6)

^1^ Only those >5 isolates were presented.

**Table 4:** Appearance of non-*Candida* species ^1^ in the oral samples on CHROMagar *Candida* plates.

Yeast Species	Isolates (Total 320)	Morphology	Colony Color	Frequency of Regular Type
*Trichosporon asahii*	18 (5.6%)	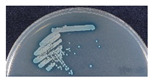	Light blue	100% (18/18)
*Cutaneotrichosporon mucoides*	6 (1.9%)	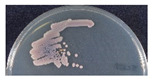	Light purple	100% (6/6)
*Saccharomyces cerevisiae*	7 (2.2%)	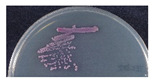	Purple	100% (7/7)
*Exophiala dermatitidis*	5 (1.6%)	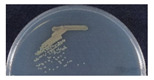	Greenish brown	100% (5/5)
*Magnusiomyces capitatus*	5 (1.6%)	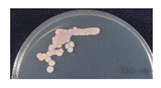	Pale pink	100% (5/5)

^1^ Only those >5 isolates were presented.

**Table 5:** Proportions of color-matched species on CHROMagar *Candida* plates.

Color Group	Isolate Number	Final ID	Regular Color	Color-Matched	% In Total (311)	% In Color Group
**Green**	130	*Candida albicans*	green	129	41.5%	99.2%
		*Candida dubliniensis*	dark green	1	0.3%	0.8%
**Purple/Pink**	90	*Candida glabrata*	purple	51	16.4%	56.7%
		*Saccharomyces cerevisiae*	purple	7	2.3%	7.8%
		*Cutaneotrichosporon mucoides*	light purple	6	1.9%	6.7%
		*Magnusiomyces capitatus*	pale pink	5	1.6%	5.6%
		*Candida pararugosa*	very light purple	4	1.3%	4.4%
		*Meyerozyma guilliermondii*	light purple	4	1.3%	4.4%
		*Pichia kudriavzevii*	purple with white border	4	1.3%	4.4%
		*Pichia manshurica*	pale pink	2	0.6%	2.2%
		*Candida ethanolica*	pale pink with white border	1	0.3%	1.1%
		*Candida fermentati*	light purple	1	0.3%	1.1%
		*Candida intermedia*	dark purple	1	0.3%	1.1%
		*Candida metapsilosis*	light purple	1	0.3%	1.1%
		*Clavispora lusitaniae*	light purple	1	0.3%	1.1%
		*Cyberlindnera jadinii*	light purple	1	0.3%	1.1%
		*Kluyveromyces marxianus*	light purple	1	0.3%	1.1%
**Blue**	42	*Candida tropicalis*	metallic blue	20	6.4%	47.6%
		*Trichosporon asahii*	blue	18	5.8%	42.9%
		*Trichosporon faecale*	blue	2	0.6%	4.8%
		*Cutaneotrichosporon jirovecii*	blue	1	0.3%	2.4%
		*Lodderomyces elongisporus*	metallic blue	1	0.3%	2.4%
**White**	38	*Candida parapsilosis*	white	32	10.3%	84.2%
		*Candida orthopsilosis*	white	3	1.0%	7.9%
		*Pichia norvegensis*	white	2	0.6%	5.3%
		*Cryptococcus neoformans*	milk white	1	0.3%	2.6%
**Gray**	7	*Exophiala dermatitidis*	olivaceous-gray	5	1.6%	71.4%
		*Fereydounia khargensis*	olivaceous-gray	2	0.6%	28.6%
**Orange**	4	*Lachancea fermentati*	red-brown	2	0.6%	50.0%
		*Rhodotorula mucilaginosa*	orange	1	0.3%	25.0%
		*Rhodotorula toruloides*	orange	1	0.3%	25.0%

## Data Availability

The data presented in this study are available on request from the corresponding author.

## Supplementary Materials

The following are available online at https://www.mdpi.com/2076-2607/9/1/142/s1, Supplementary figures contains overlaid mass spectra with smoothing and baseline subtraction for *Candida albicans* isolates (Figure S1), *Candida grablata* isolates (Figure S2), *Candida parapsilosis* isolates (Figure S3), *Candida tropicalis* isolates (Figure S4) and *Trichosporon asahii* isolates (Figure S5).
