# Supplementary material for: The Use of MALDI-TOF Mass Spectrometry to Analyze Commensal Oral Yeasts in Nursing Home Residents
Source: Microorganisms. 2021 Jan 9;9(1):142. doi: 10.3390/microorganisms9010142 (PMC7828027; doi:10.3390/microorganisms9010142)
Supplement: Supplementary file 1 [file microorganisms-09-00142-s001.pdf]

Supplementary Materials

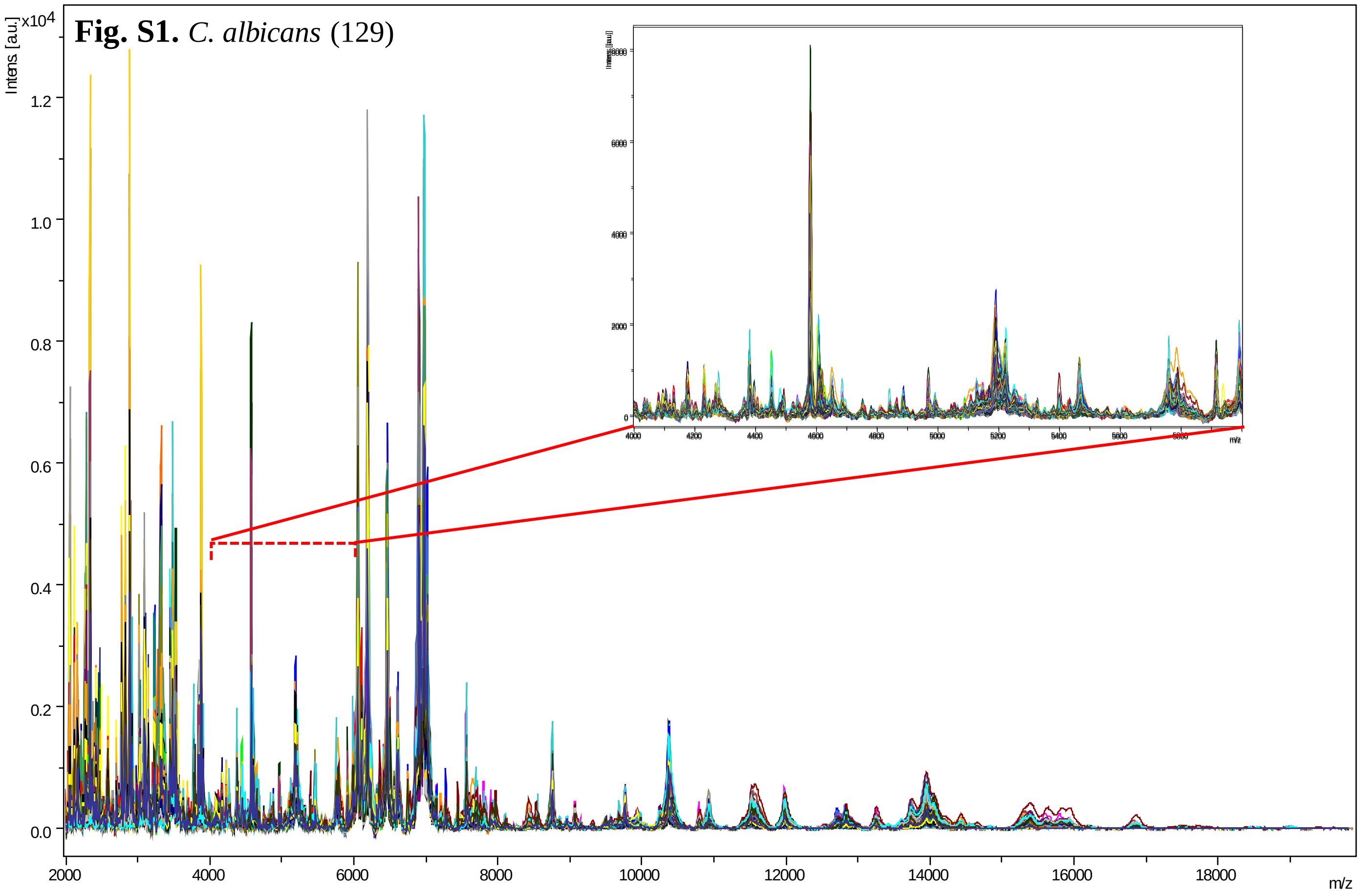

Figure S1. Overlaid mass spectra with smoothing and baseline subtraction for *Candida albicans* isolates. All the 129 isolates were identified as *C. albicans* by MALDI-TOF MS with score values higher than 2. The insert is the zoom region showing ion between m/z 4,000 and 6,000. The mass spectra were processed using the FlexAnalysis 3.4 program (Bruker Daltonics, Bremen, Germany).

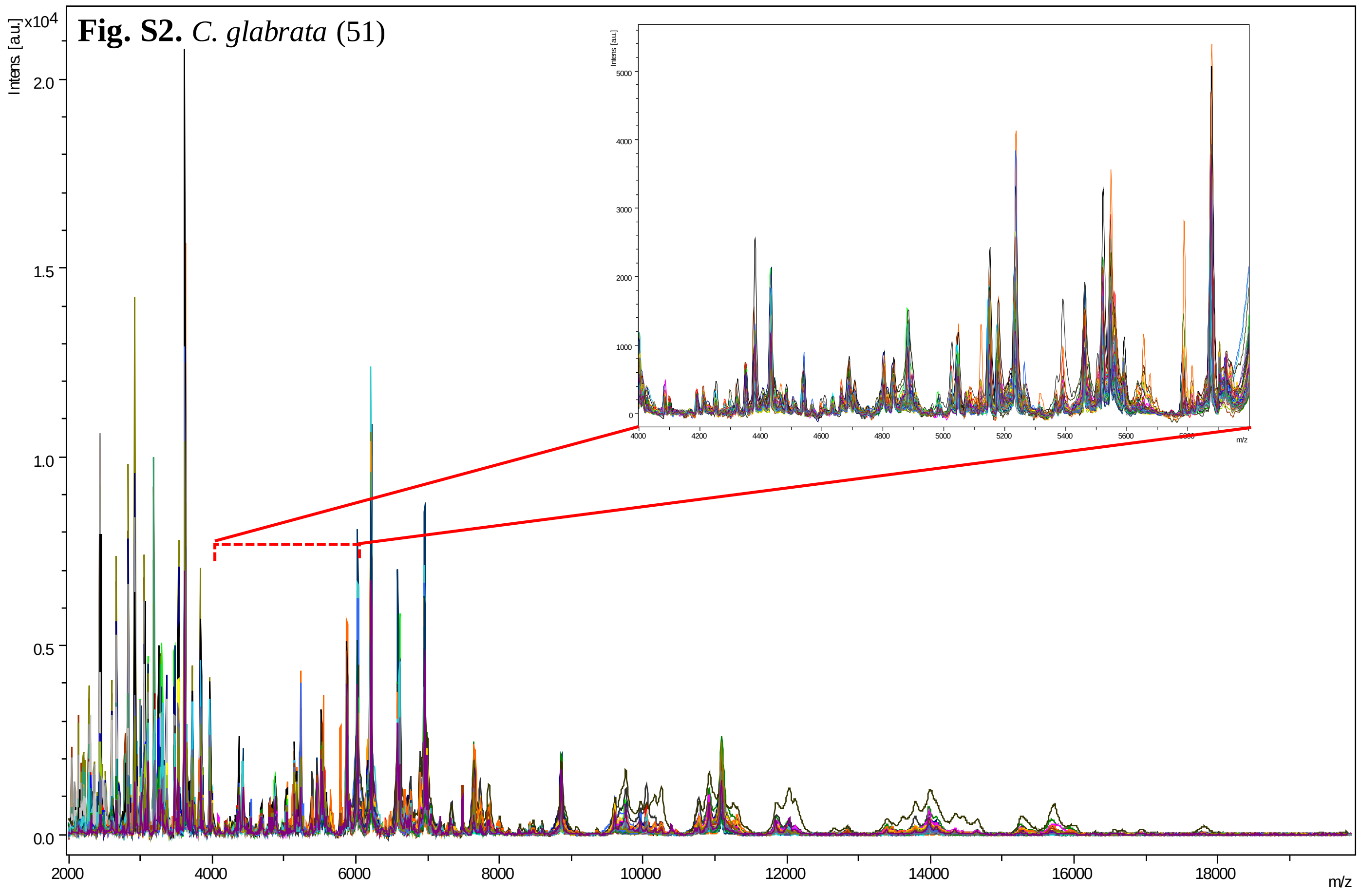

Figure S2. Overlaid mass spectra with smoothing and baseline subtraction for *Candida grablata* isolates. All the 51 isolates were identified as *C. grablata* by MALDI-TOF MS with score values higher than 2. The insert is the zoom region showing ion between m/z 4,000 and 6,000. The mass spectra were processed using the FlexAnalysis 3.4 program (Bruker Daltonics, Bremen, Germany).

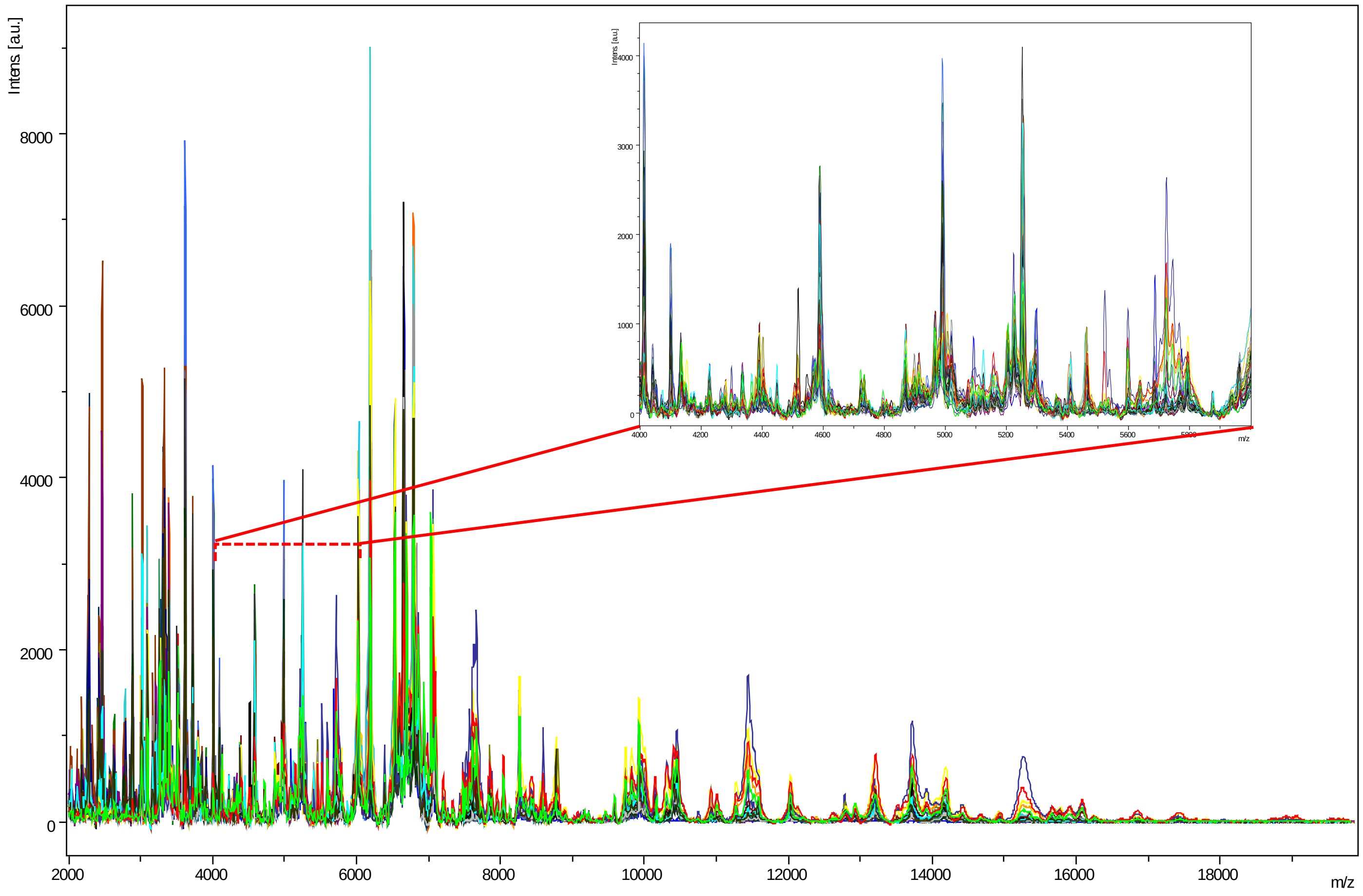

Figure S3. Overlaid mass spectra with smoothing and baseline subtraction for *Candida parapsilosis* isolates. All the 34 isolates were identified as *C. parapsilosis* by MALDI-TOF MS with score values higher than 2. The insert is the zoom region showing ion between m/z 4,000 and 6,000. The mass spectra were processed using the FlexAnalysis 3.4 program (Bruker Daltonics, Bremen, Germany).

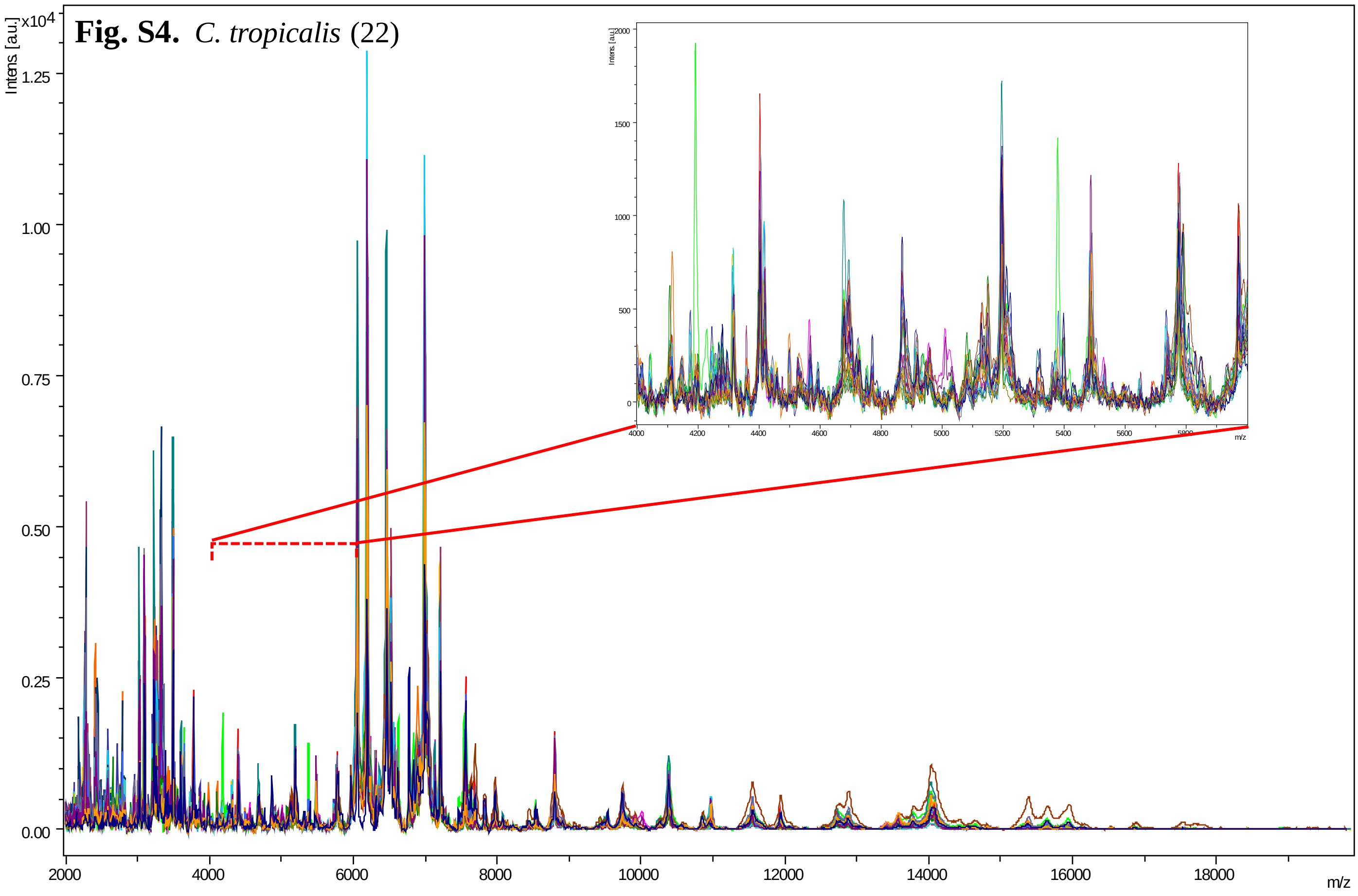

Figure S4. Overlaid mass spectra with smoothing and baseline subtraction for *Candida tropicalis* isolates. All the 22 isolates were identified as *C. tropicalis* by MALDI-TOF MS with score values higher than 2. The insert is the zoom region showing ion between m/z 4,000 and 6,000. The mass spectra were processed using the FlexAnalysis 3.4 program (Bruker Daltonics, Bremen, Germany).

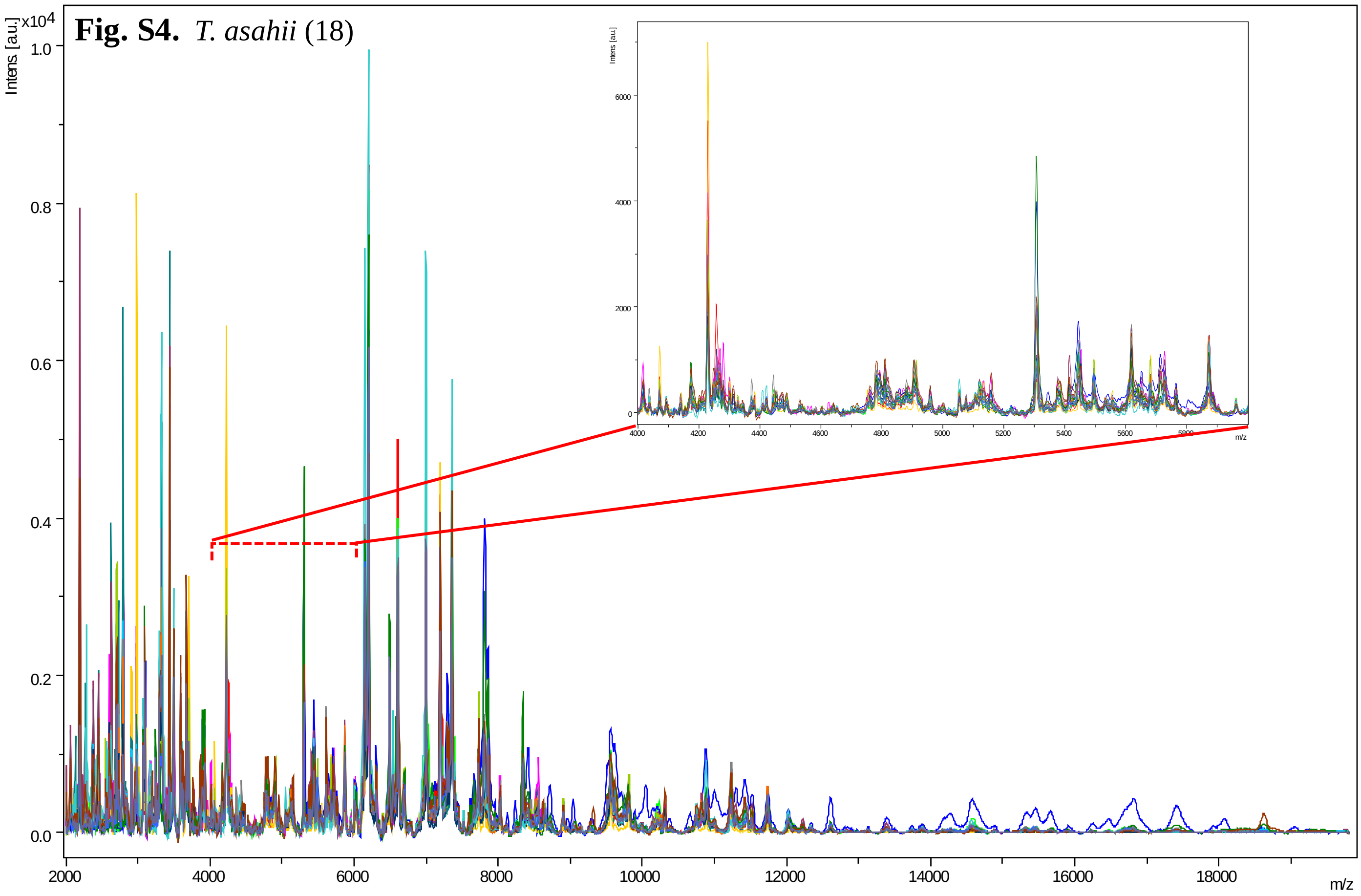

Figure S5. Overlaid mass spectra with smoothing and baseline subtraction for *Trichosporon asahii* isolates. All the 18 isolates were identified as *T. asahii* by MALDI-TOF MS with score values higher than 2. The insert is the zoom region showing ion between m/z 4,000 and 6,000. The mass spectra were processed using the FlexAnalysis 3.4 program (Bruker Daltonics, Bremen, Germany).
